# COHORT CHANGE AND LIFE COURSE SOCIOECONOMIC DIFFERENCES IN COGNITIVE HEALTH TRAJECTORIES

**DOI:** 10.1093/geroni/igad104.3234

**Published:** 2023-12-21

**Authors:** Hyungmin Cha

**Affiliations:** University of Southern California, Los Angeles, California, United States

## Abstract

The combination of socioeconomic gains and losses during a lifetime contributes to a large inequality in cognitive capacity in later life. Although studies have examined the connection between an individual’s socioeconomic status over the life course and cognitive health trajectories, it is unclear how historical contexts may have influenced these associations. In light of the dynamic macro-social environment in Korea, this study utilizes data from the Korean Longitudinal Study of Aging (KLoSA) from 2006 to 2018. I explore the variation in the socioeconomic patterning of cognitive health trajectories based on the benchmarking of socioeconomic status across the life course. The study reveals three main findings. First, I find cohort-specific cognitive health trajectories based on socioeconomic status across the life course. Notably, childhood socioeconomic status leaves an indelible mark on levels of cognition only among earlier cohorts born during the Japanese Occupation period. Additionally, socioeconomic disparities in the rate of cognitive health decline widen among earlier cohorts, while this trend is not observed among more recent cohorts. Second, compared to childhood and later-life socioeconomic status, education consistently shows a positive association with higher levels of cognition rather than slower cognitive decline, regardless of whether the cohorts are earlier or more recent. Third, there is a notable gender difference in socioeconomic disparities in cognitive decline, with women experiencing more pronounced disparities compared to men, especially among earlier cohorts. These findings underscore the significance of historical and contextual factors when examining the association between socioeconomic status and cognitive health across the life course.

